# Sociodemographic profile of elderly people cared for at a medium and high complexity hospital: a cross-sectional study, Belo Horizonte, 2015-2019

**DOI:** 10.1590/S2237-96222025v34e20240254.en

**Published:** 2025-09-08

**Authors:** Cristiano Inácio Martins, Camilla Stephane Oliveira Silva, Fernanda Gonçalves de Souza, Shirlei Moreira da Costa Faria, Karla Rona da Silva, Mirela Castro Santos Camargos

**Affiliations:** 1 Universidade Federal de Minas Gerais, Escola de Enfermagem, Programa de Pós-Graduação em Gestão de Serviços de Saúde, Belo Horizonte, MG, Brasil; 2 Universidade Federal de Minas Gerais, Escola de Enfermagem, Belo Horizonte, MG, Brasil; 3 Universidade Federal de Minas Gerais, Centro de Desenvolvimento e Planejamento Regional, Programa de Pós-Graduação em Demografia, Belo Horizonte, MG, Brasil; 4 Universidade Federal de Minas Gerais, Escola de Enfermagem, Programa de Pós-Graduação em Enfermagem, Belo Horizonte, MG, Brasil; 5 Universidade Federal de Minas Gerais, Escola de Enfermagem,Departamento de Gestão em Saúde, Belo Horizonte, MG, Brasil

**Keywords:** Hospitalization, Elderly, Emergency Medical Services, Multiple Trauma, Cross-Sectional Studies, Hospitalización, Anciano, Servicios Médicos de Emergencia, Traumatismo Múltiple, Estudios Transversales

## Abstract

**Objective:**

To analyze the sociodemographic profile of elderly individuals hospitalized in a medium and high complexity hospital in Belo Horizonte, with emphasis on reasons for hospitalization, length of hospital stay, and factors associated with risk of death.

**Methods:**

This is a descriptive, quantitative, cross-sectional study based on data from electronic medical records of elderly individuals (≥60 years) treated between 2015 and 2019 at a referral hospital for multiple trauma in Belo Horizonte. The variables investigated included age, sex, marital status, municipality of origin, reason for hospitalization, and length of stay. Descriptive statistical analysis, Kaplan-Meier estimator, log-rank test, and Cox regression were used to identify association between the factors analyzed.

**Results:**

A total of 10,742 medical records were analyzed, the majority of which were male (52.6%) and aged 60-79 years (75.8%). The most frequent reason for hospitalization was falls (44.4%), followed by clinical causes (25.1%). Average length of hospital stay was 79.8 days. The mortality rate was 11.7%, with higher risk associated with self-harm (3.1 times higher), falls and poisoning (1.9 times higher). The longest hospital stay was observed in cases of people who had been run over (96 days).

**Conclusions:**

The profile found highlighted the vulnerability of the elderly population, the complexity of the cases treated and the need for specific hospital management strategies. The importance of preventive actions, palliative care and continuous training of professionals stood out, with the aim of improving the quality of life of the elderly population and optimizing health resources.

## Resumen

**Objetivo:**

Analizar el perfil sociodemográfico de los ancianos hospitalizados en un hospital de media y alta complejidad de Belo Horizonte, con énfasis en los motivos de hospitalización, tiempo de internación y factores asociados al riesgo de muerte.

**Métodos:**

Se trata de un estudio descriptivo, cuantitativo, transversal, realizado con base en datos de historias clínicas electrónicas de ancianos (≥60 años) atendidos entre 2015 y 2019 en un hospital de referencia para traumatismo múltiple de Belo Horizonte. Las variables investigadas incluyeron edad, sexo, estado civil, municipio de procedencia, motivo de hospitalización y duración de la estancia hospitalaria. Se utilizó análisis estadístico descriptivo, estimador de Kaplan-Meier, prueba log-rank y regresión de Cox para identificar asociaciones entre los factores analizados.

**Resultados:**

Se analizaron 10.742 historias clínicas, la mayoría fueron de sexo masculino (52,6%) y en el rango de edad de 60-79 años (75,8%). El motivo más frecuente de hospitalización fueron las caídas (44,4%), seguido de las causas clínicas (25,1%). La duración media de la estancia hospitalaria fue de 79,8 días. La tasa de mortalidad fue del 11,7%, con un mayor riesgo asociado a las autolesiones (3,1 veces mayor), las caídas y las intoxicaciones (1,9 veces). La estancia hospitalaria más larga se observó en los casos de accidentes por atropello (96 días).

**Conclusiones:**

El perfil destacó la vulnerabilidad de la población anciana, la complejidad de los casos atendidos y la necesidad de estrategias específicas de gestión hospitalaria. Se destacó la importancia de las acciones preventivas, cuidados paliativos y formación continua de los profesionales, visando mejorar la calidad de vida de la población anciana y optimizar los recursos de salud.

**Ethical aspects**

This research respected ethical principles, having obtained the following approval data:

Research Ethics Committee: Universidade Federal de Minas Gerais

Opinion number: 3,082,692

Approval date: 13/12/2018

Certificate of Submission for Ethical Appraisal: 98627418.0.0000.5149

Informed Consent Form: Exempt.

## Introduction

Over the years, the demographic dynamics of the Brazilian population have undergone significant changes, particularly with regard to age structure. The reduction in the birth rate has resulted in an increase in the number of elderly people in Brazil when compared to the total population of the country in the period 2012-2022, which increased from 11.3% to 15.1% (1).

This demographic change raises a series of crucial questions that impact the population dynamics and the epidemiological profile of the country. This scenario highlights the need for strategic planning of public policies, with a focus on public health, to meet the growing demands of the elderly population at various levels of care and prevention (2).

Since 2003, actions aimed at protecting and promoting the rights of the elderly have been implemented as public policies, as a result of the enactment of the Elderly Persons Statute. This legal move forward is considerable, since it assists and promotes priority access to fundamental rights for all people aged 60 or over. In 2006, the National Health Policy for the Elderly reinforced the guarantee of rights through collective and individual health measures recommended by various guidelines (3,4).

Comprehensive health care for the elderly seeks to address the particularities of senescence and senility. Senescence is characterized by a cumulative, irreversible loss of functional reserve associated with aging, which although not a disorder, it can become one, depending on stimuli and social determinants (4). With regard to senility, the individual presents weakness or mental illness due to old age and is associated with the deterioration of the body and mind in elderly people (5).

Promoting active and healthy aging, offering comprehensive and integrated health care for the elderly and encouraging intersectoral actions, in order to achieve comprehensive care and the provision of resources capable of ensuring quality health care for the elderly, are essential strategies for guaranteeing the quality of care and protection of the health rights of this group (4).

The medium and high complexity sectors have presented particular demands for the treatment and management of health problems. These range from neurobiological factors and mental disorders to complications arising from chronic non-communicable diseases. Such problems often result in hospitalizations (6).

Hospitalization of elderly people is permeated by several determinants, such as high cost of care, since it requires multidisciplinary and interdisciplinary care with a focus on specialties and provision of hard technologies. It should be considered that they not arise in a context of unplanned hospitalization, but rather in a context of urgent complications, with health problems with longer recovery times, requiring longer hospital stays. In many cases, the patient is readmitted due to complications arising from the underlying cause of hospitalization (7).

This study sought to analyze the sociodemographic profile of elderly people hospitalized in a medium and high complexity hospital in Belo Horizonte. Analysis of these indicators was relevant, as it allows health service managers to understand the profile of people who use the services, with ways to support the effective allocation of health resources to meet demand.

## Methods

### Design

This is a descriptive, cross-sectional study of a quantitative nature.

### Setting

The study was conducted based on data from a referral emergency service for multiple trauma in Belo Horizonte. It operates continuously with a capacity of 295 beds and, in exceptional or extreme situations, can incorporate an additional 64 emergency beds, integrating it as a Specialized Center in the Emergency Care Network. The data were collected from the database of the Integrated Hospital Management System based on electronic medical records.

### Participants

The study included individuals aged 60 years or older who were treated in a referral emergency service for multiple trauma in Belo Horizonte between 2015 and 2019. The eligibility criterion was participants aged 60 years or older, since this is the age definition of an elderly person, according to Brazilian legislation. The data source was secondary, with data obtained from the electronic medical records available in the database of the institutional Integrated Hospital Management System (3).

### Variables

The variables investigated were: age, sex, municipality, marital status, reason for hospitalization and length of stay in the unit. 

### Data sources

The data were stored on an Excel 2010 spreadsheet, coded, and a data dictionary was created, which was transcribed using Microsoft Excel spreadsheets. A numerical system was adopted for coding purposes to enable categorization. A numerical system was also used to identify the patients’ personal data. After reviewing and standardizing the codes, the data were analyzed using the Statistical Package for the Social Sciences software, version 24.

The study involved analysis of six variables, which were extracted from the same data source. The age variable analyzed the medical records of patients aged 60 years or older. The gender variable was recorded as “male” or “female,” as reported in the medical records. Marital status was classified into categories, such as “single,” “married/stable union/cohabiting,” “widowed,” and “divorced/separated.” The reason for hospitalization was identified based on the diagnoses recorded on the system, and was categorized according to the codes established by the International Classification of Diseases (ICD), 10th revision. Length of stay in the unit was calculated based on the dates of admission and discharge recorded in the medical records.

### Bias 

In order to ensure the validity and reliability of this descriptive cross-sectional study, measures were implemented to avoid potential biases that could compromise the results. Selection bias was avoided by including all available medical records that met the previously clearly established criteria. Information bias was controlled by standardizing data collection, using a form that recorded the variables of interest in a uniform manner. No significant confounding variables that could interfere with the associations evaluated in this study were identified.

### Study size 

The sample size was defined based on criteria of representativeness and data availability, considering the population of elderly people treated at a medium and high complexity hospital in Belo Horizonte between 2015 and 2019. A total of 10,742 participants were included, selected according to inclusion criteria. The choice of this sample size allowed for statistically significant analysis of the data, ensuring reliability of the results.

### Statistical methods

The description of the data was presented in the form of observed frequency, percentage, lower and upper values, and measures of central tendency and variability. The nonparametric Kaplan-Meier estimator was used to observe the event of failure (death) (8). The log-rank test was used to compare the equalness of survival curves, suggesting the existence or not of association between the independent and dependent variables (9). Cox regression was used to compare length of hospital stay until the occurrence of failure associated with possible risk factors (10). The alpha level of significance used in all the analyses was 5.0%.

This statistical approach provided more robust and detailed analyses, since it allowed the inclusion of participants from the beginning of data collection, without the need for a uniform time interval for all, as is the case in this research. Average length of hospital stay until discharge or death was examined, this being a variable that naturally changes among elderly patients. 

## Results

The results show (Table 1) that most hospitalizations were for male patients (n=5,656, 52.6%). The highest number of hospitalizations was in the 60-79 years age group (n=8,145, 75.8%), most of whom came from Belo Horizonte, where the hospitalization unit is located (n=6,849; 64.0%). The marital status of the majority was married/stable union/cohabiting (n=4,710, 57.1%). The year with the most hospitalizations was 2019 (n=2,222, 20.6%). The year with the lowest number of hospitalizations was 2015 (2,005, 18.6%).

**Table 1:** Participants by study year and study population sociodemographic data. Belo Horizonte, 2015-2019 (n=10,742)

Variables		n (%)
Year	2015	2,005 (18.6)
	2016	2,218 (20.6)
	2017	2,172 (20.2)
	2018	2,125 (19.7)
	2019	2,222 (20.6)
Sex	Male	5,656 (52.6)
	Female	5,086 (47.3)
**Age group** (years)	60-79	8,145 (75.8)
	≥80	2,597 (24.1)
Municipality	Other	3,847 (35.9)
	Belo Horizonte	6,849 (64.0)
**Marital status**	Single	1,679 (20.3)
	Widowed	1,253 (15.2)
	Married/stable union/cohabiting	4,710 (57.1)
	Divorced/separated	601 (7.2)

We found that falls were the reason for hospitalization of most of the elderly people studied (n=4,773), corresponding to 44.4% of the sample “n”, followed by clinical causes (n=2,698, 25.1%) and being run over (n=1,098, 10.2%). The other reasons for hospitalization were described in decreasing order: readmission (n=783, 7.2%), accident (n=740, 6.8%), assault (n=182, 1.6%), burn (n=179; 1.6%), poisoning (n=149, 1.3%), self-harm (n=84, 0.5%) and foreign bodies (n=56, 0.5%). Fifty percent of the patients were hospitalized for an average of 79.8 days, with a standard error (σ) of 1.8 months. Of the total number of patients analyzed, 1,255 (11.7%) events (death) were observed and the remaining 9,487 patients presented a censoring proportion of 88.3%.

**Figure 1:**
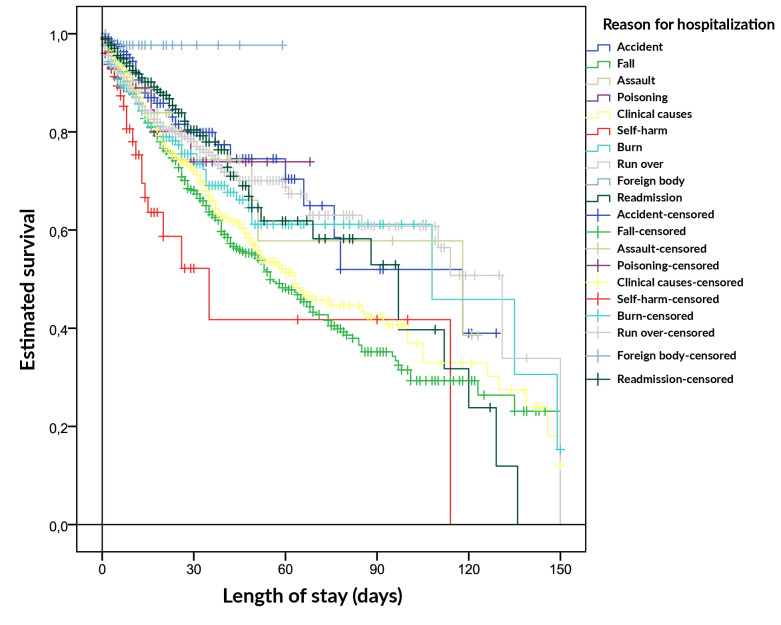
Survival curve estimated using the Kaplan-Meier method for length of hospital stay by reasons for hospitalization. Belo Horizonte, 2015-2019 (n=10,742).

The lowest proportion of failures was observed when foreign bodies were the reason for hospitalization (1.8%). The log-rank test was significant: there was a difference between the survival curves of the reasons, in which the longest estimate of hospitalization time was when the reason for hospitalization was having been run over (96 days) (Table 2 and Figure 1). 

**Table 2:** Estimate and 95% confidence interval (95%CI) of the reason for hospitalization using Kaplan-Meier survival analysis with log-rank test. Belo Horizonte, 2015-2019 (n=10,742)

Reason for hospitalization	Estimate	Standard error	95%CI	Failures n (%)	p-value (log-rank)^a^
Accident	86.73	6.92	73.16; 100.3	38 (5.1)	<0.001
Fall	71.44	2.89	65.77; 77.11	522 (10.9)	
Assault	82.29	9.33	64; 100.58	20 (11)	
Poisoning	53.84	4.17	45.66; 62.01	15 (10.1)	
Clinical causes	76.23	3.51	69.34; 83.12	368 (13.6)	
Self-harm	57.05	11.09	35.32; 78.78	21 (25)	
Burn	91.33	7.41	76.8; 105.87	52 (29.1)	
Run over	96.02	5.12	85.99; 106.05	142 (12.9)	
Foreign body	57.67	1.31	55.11; 60.24	1 (1.8)	
Readmission	81.06	5.66	69.97; 92.15	76 (9.7)	

^a^Significant when p-value <0.050.

**Figure 2:**
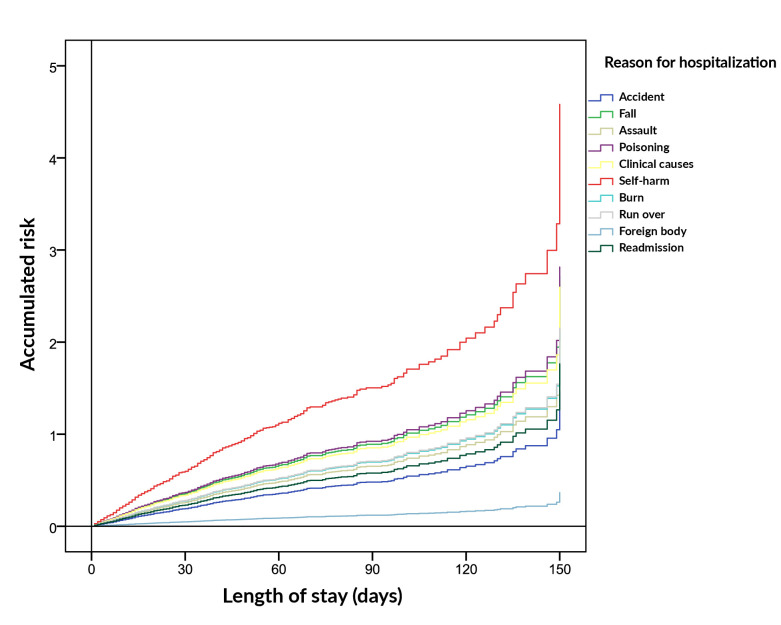
Risks estimated using the Cox method for length of hospital stay by reasons for hospitalization. Belo Horizonte, 2015-2019 (n=10.742).

The model was significant, p-value<0.001. There were differences in the risks of death between the reasons for hospitalization. Risk of death due to falls, poisoning, clinical reasons, self-harm or being run over were 1.9, 1.9, 1.8, 3.1 and 1.5 times higher in relation to accidents as the reason for hospitalization (Table 3 and Figure 2).

**Table 3:** Risk ratios (RR) and 95% confidence intervals (95%CI) of the reason for hospitalization using Cox regression. Belo Horizonte, 2015-2019 (n=10,742)

Reason for hospitalization	RR	95%CI	p-value
Accident	1	-	-
Fall	1.856	1.335; 2.581	<0.001
Assault	1.358	0.790; 2.334	0.269
Poisoning	1.925	1.059; 3.499	0.032
Clinical causes	1.775	1.271; 2.480	0.001
Self-harm	3.133	1.838; 5.341	<0.001
Burn	1.453	0.954; 2.212	0.082
Run over	1.471	1.028; 2.105	0.035
Foreign body	0.250	0.034; 1.818	0.171
Readmission	1.205	0.816; 1.780	0.347

The lower proportion of failures associated with hospitalization due to foreign bodies (1.8%) highlighted the possible relationship between this reason for hospitalization and better clinical results. This phenomenon suggested a less severe condition or lower risk of adverse outcomes.

The relevance of the log-rank test, which indicated significant differences in the survival curves in relation to the different reasons for hospitalization and the longest estimated hospitalization time due to being run over (96 days), highlights the need for a personalized approach for each case. The variation in the risks of death associated with the different reasons for hospitalization (1.9, 1.9, 1.8, 3.1 and 1.5 times higher) emphasized the need for specific assessments and care for each situation.

## Discussion

When analyzing prevalence of hospitalizations of elderly people, we identified that the male sex was predominant, which highlighted the greater vulnerability of elderly men, especially in situations involving external accidents or worsening of chronic clinical conditions. The age groups corresponded to elderly people aged 60-64 and 65-69 years, possibly due to the greater degree of independence and involvement in physical and social activities, which can increase exposure to trauma. We found that most hospitalizations occurred in Belo Horizonte, a fact related to the location of the reference hospital in the capital of the state of Minas Gerais and the population context of a city with more than 2.5 million inhabitants (11). This demonstrated that the provision of health services in Minas Gerais occurs in accordance with the Health Regionalization Master Plan, assigning to each region a share of responsibility for care according to economy of scale and scope in order to increase accessibility to the treatment required.

The study showed that 50.0% of hospitalized elderly individuals remained hospitalized for an average of 79.8 days, a finding that reinforces the importance of strategies aimed at efficiently managing hospitalization time. The 11.7% mortality rate observed, together with the clinical challenges highlighted during follow-up, highlight the complexity of the cases treated and point to the need to strengthen palliative care and emotional support. These results revealed a panorama that demands interdisciplinary efforts to improve the quality of life of the elderly population, reduce the costs associated with prolonged hospital care, and offer more appropriate care alternatives.

The results reveal a series of significant implications for health services, especially when considering the increase in length of stay and the gradual reduction in patient survival rates. The fact that 50.0% of elderly people remained hospitalized for an average of 79.8 days, with a standard deviation of σ of 1.8 months, indicates a considerable average length of hospital stay. Lengthy stays can lead to high costs for health services, including the use of financial resources, prolonged occupation of hospital beds and increased demand on medical staff (12,13).

The 11.7% mortality rate and the 88.3% censoring rate for the remaining patients highlights the complexity of the cases treated. These indicators suggest a challenging clinical scenario, in which many people are unable to complete the follow-up they need. This context may be associated with the difficulty in maintaining the elderly population under intensive care for prolonged periods, highlighting the importance of strategies aimed at palliative care and emotional support (14).

For health services, these findings point to the need to implement more efficient management strategies that include reducing hospital stays, improving quality of life, and offering alternative care options for the elderly population. Collaboration between multidisciplinary teams, attention to factors that influence survival rates, and optimization of resource use are essential to ensure a balance between clinical effectiveness, patient quality of life, and sustainability of health services (12,15).

The risk analysis estimated by the Cox method, regarding the length of hospital stay according to the reasons for hospitalization, demonstrated the relevance of effective triage at the time of hospital admission. This practice allows for the most appropriate allocation of resources and the application of targeted interventions. It is noteworthy that the quality of life of the elderly was twice as high with health care strategies specific to the reasons for hospitalization. By adapting treatment and prevention approaches, health services can improve clinical outcomes and contribute to a better quality of life for the elderly, addressing their needs in a comprehensive and efficient manner (3,16,17).

This study provided a holistic perspective on health services and the quality of life of the elderly population, addressing aspects such as length of hospital stay, risk of death, gender, age group, and reason for hospitalization. This multifaceted approach highlighted the complexity of care for the elderly and highlighted the importance of integrated and personalized intervention to optimize health outcomes and quality of life in this demographic group. The importance of implementing public policies to prevent trauma and clinical problems in the elderly population was made evident. 

Through implementation of measures that promote healthy aging, prevention and effective treatment of chronic diseases, in addition to traffic education campaigns, it is possible to achieve significant results in improving the quality of life of the elderly. It is important to highlight the importance of health professionals working in this context being continuously trained to offer the most qualified care and treatment to the elderly population.

## Data Availability

The database is available upon request from the peer reviewers, upon plausible justification, given that it contains sensitive data.
