# Supplementary material for: Sociodemographic profile of elderly people cared for at a medium and high complexity hospital: a cross-sectional study, Belo Horizonte, 2015-2019
Source: Epidemiol Serv Saude. 2025 Sep 8;34:e20240254. doi: 10.1590/S2237-96222025v34e20240254.en (PMC12435965; doi:10.1590/S2237-96222025v34e20240254.en)
Supplement: Translation

# Translation: Perfil sociodemográfico de pessoas idosas atendidas em um hospital de média e alta complexidade: estudo transversal, Belo Horizonte, 2015-2019

**DOI:** 10.1590/S2237-96222025v34e20240254.pt

**Authors:** Cristiano Inácio Martins, Camilla Stephane Oliveira Silva, Fernanda Gonçalves de Souza, Shirlei Moreira da Costa Faria, Karla Rona da Silva, Mirela Castro Santos Camargos

**Affiliations:** 1 Universidade Federal de Minas Gerais, Escola de Enfermagem, Programa de Pós-Graduação em Gestão de Serviços de Saúde, Belo Horizonte, MG, Brasil; 2 Universidade Federal de Minas Gerais, Escola de Enfermagem, Belo Horizonte, MG, Brasil; 3 Universidade Federal de Minas Gerais, Centro de Desenvolvimento e Planejamento Regional, Programa de Pós-Graduação em Demografia, Belo Horizonte, MG, Brasil; 4 Universidade Federal de Minas Gerais, Escola de Enfermagem, Programa de Pós-Graduação em Enfermagem, Belo Horizonte, MG, Brasil; 5 Universidade Federal de Minas Gerais, Escola de Enfermagem,Departamento de Gestão em Saúde, Belo Horizonte, MG, Brasil

**Keywords:** Hospitalização, Pessoa Idosa, Serviços Médicos de Emergência, Traumatismo Múltiplo, Estudos Transversais

## Abstract

**Objetivo:**

Analisar o perfil sociodemográfico de pessoas idosas hospitalizadas em um hospital de média e alta complexidade em Belo Horizonte, com ênfase nos motivos de internação, tempo de permanência hospitalar e fatores associados ao risco de morte.

**Métodos:**

Trata-se de estudo transversal descritivo, quantitativo, realizado com base em dados de prontuários eletrônicos de pessoas idosas (≥60 anos) atendidas entre 2015 e 2019 em um hospital de referência para politraumatismo em Belo Horizonte. As variáveis investigadas incluíram idade, sexo, estado civil, município de origem, motivo de internação e período de permanência. Foram utilizados análise estatística descritiva, estimador de Kaplan-Meier, teste de log-rank e regressão de Cox para identificar associações entre os fatores analisados.

**Resultados:**

Analisaram-se 10.742 prontuários, sendo a maioria do sexo masculino (52,6%) e na faixa etária de 60-79 anos (75,8%). O motivo mais frequente de internação foi queda (44,4%), seguido de causas clínicas (25,1%). O tempo médio de internação foi 79,8 dias. A taxa de óbitos foi 11,7%, com maior risco associado à autoagressão (3,1 vezes maior), quedas e intoxicação (1,9 vez). O maior tempo de internação foi observado em casos de atropelamento (96 dias).

**Conclusões:**

O perfil evidenciado destacou a vulnerabilidade da população idosa, a complexidade dos casos atendidos e a necessidade de estratégias específicas de gestão hospitalar. Enfatizou-se a importância de ações preventivas, cuidados paliativos e capacitação contínua dos profissionais, visando à melhoria da qualidade de vida da população idosa e à otimização dos recursos em saúde.

**Aspectos éticos**

A presente pesquisa respeitou os princípios éticos, obtendo os seguintes dados de aprovação:

Comitê de ética em pesquisa: Universidade Federal de Minas Gerais

Número do parecer: 3.082.692

Data de aprovação: 13/12/2018

Certificado de apresentação de apreciação ética: 98627418.0.0000.5149

Registro do consentimento livre e esclarecido: Dispensado.

## Introdução

Com o passar dos anos, a dinâmica demográfica da população brasileira passou por transformações significativas, particularmente no que diz respeito à estrutura etária. A redução da taxa de natalidade populacional resultou no aumento de pessoas idosas no país quando comparado à população total do Brasil no período 2012-2022, que passou de 11,3% para 15,1% (1).

Essa mudança demográfica suscita uma série de questões cruciais que impactam na dinâmica populacional e no perfil epidemiológico do país. Esse cenário evidencia a necessidade de planejamento estratégico de políticas públicas, com foco na área de saúde, para atender às crescentes demandas da população idosa nos variados níveis de atenção e prevenção (2).

Desde 2003, ações voltadas à proteção e promoção dos direitos da pessoa idosa têm sido efetivadas como políticas públicas, em decorrência da promulgação do Estatuto da Pessoa Idosa. Esse avanço legal é considerável, uma vez que auxilia e promove a todas as pessoas com 60 anos ou mais prioridade ao acesso aos direitos fundamentais. Em 2006, a Política Nacional de Saúde da Pessoa Idosa reforçou a garantia dos direitos por meio de medidas coletivas e individuais de saúde preconizadas por diversas diretrizes (3,4).

A atenção integral à saúde da pessoa idosa busca abordar as particularidades da senescência e da senilidade. A senescência é caracterizada por perda de reserva funcional cumulativa, irreversível, associada ao envelhecimento, porém não patológica, mas a depender dos estímulos e dos determinantes sociais, pode se tornar uma patologia (4). Na senilidade, o indivíduo se apresenta com fraqueza ou enfermidade mental da velhice e associa-se à deterioração do corpo e da mente nas pessoas idosas (5). Promover o envelhecimento ativo e saudável, oferecer atenção integral e integrada à saúde da pessoa idosa e incentivar ações intersetoriais, de forma a alcançar a integralidade da atenção e o provimento de recursos capazes de assegurar qualidade da atenção à saúde da pessoa idosa, são estratégias essenciais para garantir a qualidade da atenção e proteção dos direitos à saúde desse grupo (4).

Os setores da média e alta complexidade têm apresentado particularmente demandas para o tratamento e manejo de agravos à saúde. Estes vão desde fatores neurobiológicos e transtornos mentais até complicações decorrentes de doenças crônicas não transmissíveis. Tais agravos frequentemente resultam em internações hospitalares (6).

A internação hospitalar de pessoas idosas é permeada por diversos determinantes a exemplo do alto custo assistencial, visto que demanda assistência multi e interdisciplinar com foco nas especialidades e disponibilização de tecnologias duras. Deve-se considerar que emergem em um contexto de não planejamento da internação e, sim, de intercorrência urgente, com agravos cujo tempo de recuperação é maior, exigindo maior tempo de internação. Em muitos casos, ocorre a readmissão do paciente em decorrência de agravos provenientes de complicações da causa base da internação (7).

Este estudo buscou analisar o perfil sociodemográfico das pessoas idosas hospitalizadas em um hospital de média e alta complexidade em Belo Horizonte. A análise desses indicadores foi relevante, pois permite aos gestores o conhecimento do perfil do usuário que utiliza o serviço, com vias a subsidiar em evidências a alocação efetiva de recursos em saúde para atender à demanda.

## Métodos

### Desenho do estudo

Trata-se de estudo transversal descritivo, de natureza quantitativa. 

### Contexto 

Realizou-se o estudo a partir dos dados de atendimento de um pronto-socorro de referência para politraumatismo de Belo Horizonte. Funciona ininterruptamente com capacidade operacional de 295 leitos podendo, em situação de excepcionalidade ou de extrema necessidade, incorporarem mais 64 leitos emergenciais integrando como Centro Especializado Rede de Atenção às Urgências e Emergências. A coleta de dados ocorreu no banco de dados do Sistema Integrado de Gestão Hospitalar por meio do prontuário eletrônico. 

### Participantes

Participaram do estudo pessoas com idade superior ou igual a 60 anos, atendidas no período 2015-2019 em um pronto-socorro referência para politraumatismo de Belo Horizonte. O critério de elegibilidade foi participantes com idade maior ou igual a 60 anos, uma vez que essa é a definição etária de pessoa idosa, conforme a legislação brasileira. A fonte para os dados foi secundária, sendo os dados obtidos no prontuário eletrônico disponível no banco de dados do Sistema Integrado de Gestão Hospitalar institucional (3).

### Variáveis

As variáveis investigadas foram: idade, sexo, município, estado civil, motivo da internação e período de permanência na unidade.

### Fontes de dados

Os dados foram armazenados em uma planilha do programa Excel 2010, codificados, sendo elaborado um dicionário de dados, os quais foram transcritos utilizando-se planilhas do aplicativo Microsoft Excel. Para a codificação, foi adotado um sistema numérico com o propósito de categorização. Para a identificação dos dados pessoais dos pacientes, também foi empregado um sistema numérico. Após revisão e padronização dos códigos, ocorreu a análise dos dados no software Statistical Package for the Social Sciences versão 24. 

O estudo contou com seis variáveis analisadas, as quais foram extraídas da mesma fonte de dados. A variável idade analisou os prontuários de pacientes com 60 anos ou mais. A variável sexo foi registrada como “masculino” ou “feminino”, conforme informado no prontuário. Estado civil foi classificado em categorias, sendo “solteiro”, “casado(a)/união estável/amigado”, “viúvo” e “divorciado/separado”. Motivo de internação foi identificado a partir dos diagnósticos registrados no sistema, sendo categorizado de acordo com os códigos estabelecidos pela Classificação Internacional de Doenças (CID), 10ª revisão. Período de permanência na unidade foi calculado com base nas datas de admissão e alta registradas nos prontuários. 

### Viés 

A fim de garantir a validade e confiabilidade deste estudo transversal descritivo, foram implementadas medidas para evitar potenciais vieses que pudessem comprometer os resultados. O viés de seleção foi evitado pela inclusão de todos os prontuários disponíveis que atendiam aos critérios estabelecidos de forma clara previamente. O viés de informação foi controlado por meio da padronização da coleta de dados, utilizando um formulário que registrou as variáveis de interesse de maneira uniforme. Não foram identificadas variáveis de confusão significativas que pudessem interferir nas associações avaliadas neste estudo. 

### Tamanho do estudo 

O tamanho da amostra foi definido com base em critérios de representatividade e disponibilidade de dados, considerando a população de pessoas idosas atendidas em um hospital de média e alta complexidade em Belo Horizonte entre 2015 e 2019. Foram incluídos 10.742 participantes, selecionados conforme critérios de inclusão. A escolha desse tamanho amostral permitiu a análise estatisticamente significativa dos dados, garantindo confiabilidade nos resultados. 

### Métodos estatísticos

A descrição dos dados foi apresentada na forma de frequência observada, porcentagem, valores mínimo e máximo, medidas de tendência central e de variabilidade. Utilizou-se o estimador não paramétrico de Kaplan-Meier para observar o evento de falha (óbito) (8). O teste de log-rank foi utilizado para comparar a igualdade das curvas de sobrevida sugerindo a existência ou não de associação entre as variáveis independentes e a dependente (9). A regressão de Cox foi utilizada para comparar o tempo de internação até a ocorrência do evento de falha associado aos possíveis fatores de risco (10). O nível alfa de significância utilizado em todas as análises foi 5,0%.

Essa abordagem estatística proporcionou análises mais robustas e detalhadas, visto que permitiu a inclusão de participantes desde o início da coleta de dados, sem a necessidade de intervalo de tempo uniforme para todos, como é o caso desta pesquisa. Foi examinado o tempo médio de internação hospitalar até a alta ou o óbito, variável que naturalmente muda entre os pacientes da população idosa. 

## Resultados

Os resultados demonstram (Tabela 1) que a maior parte das internações ocorreu com pacientes do sexo masculino (n=5.656, 52,6%). O maior número de internações foi na faixa etária de 60-79 anos (n=8.145, 75,8%) em sua maioria provenientes de Belo Horizonte onde está localizada a unidade de internação (n=6.849; 64,0%). O estado civil da maioria foi casado(a)/união estável/amigado (n=4.710, 57,1%). O ano em que ocorreu mais internação foi 2019 (n=2.222, 20,6%). O ano com menor número de internações foi 2015 (2.005, 18,6%).

**Tabela 1:** Participantes por ano de estudo e dados sociodemográficos da população de estudo. Belo Horizonte, 2015-2019 (n=10.742)

**Variáveis**		n (%)
Ano	2015	2.005 (18,6)
	2016	2.218 (20,6)
	2017	2.172 (20,2)
	2018	2.125 (19,7)
	2019	2.222 (20,6)
Sexo	Masculino	5.656 (52,6)
	Feminino	5.086 (47,3)
**Faixa etária** (anos)	60-79	8.145(75,8)
	≥80	2.597 (24,1)
Município	Outros	3.847 (35,9)
	Belo Horizonte	6.849 (64,0)
**Estado civil**	Solteiro(a)	1.679 (20,3)
	Viúvo(a)	1.253 (15,2)
	Casado(a)/união estável/amigado	4.710 (57,1)
	Divorciado(a)/separado(a)	601 (7,2)

Verificou-se que a maior parte da população idosa teve como motivo de entrada quedas (n=4.773) correspondendo a 44,4% do “n” amostral, seguido de motivo clínico (n=2.698, 25,1%) e atropelamento (n=1.098, 10,2%). Os demais motivos de entrada foram descritos de forma decrescente: retorno (n=783, 7,2%), acidente (n=740, 6,8%), agressão (n=182, 1,6%), queimadura (n=179; 1,6%), intoxicação (n=149, 1,3%), autoagressão (n=84, 0,5%) e corpo estranho (n=56, 0,5%). Cinquenta por cento dos pacientes ficaram internados em média 79,8 dias com erro-padrão (σ) de 1,8 mês. Do total de pacientes analisados, foram observados 1.255 (11,7%) eventos (óbito) e os demais 9.487 pacientes apresentaram a proporção de censura de 88,3%. 

A menor proporção de falhas foi observada para o motivo de entrada por corpo estranho (1,8%). O teste de log-rank foi considerável: houve diferença entre as curvas de sobrevida dos motivos, em que a maior estimativa de tempo de internação foi para o motivo de atropelamento (96 dias) (Tabela 2 e Figura 1).

**Tabela 2:** Estimativa e intervalo de confiança de 95% (IC95%) do motivo de entrada no hospital via análise de sobrevida Kaplan-Meier com teste de log-rank. Belo Horizonte, 2015-2019 (n=10.742)

Motivo de entrada	Estimativa	Erro-padrão	IC95%	Falhas n (%)	p-valor (log-rank)^a^
Acidente	86,73	6,92	73,16; 100,3	38 (5,1)	<0,001
Queda	71,44	2,89	65,77; 77,11	522 (10,9)	
Agressão	82,29	9,33	64; 100,58	20 (11)	
Intoxicação	53,84	4,17	45,66; 62,01	15 (10,1)	
Motivo clínico	76,23	3,51	69,34; 83,12	368 (13,6)	
Autoagressão	57,05	11,09	35,32; 78,78	21 (25)	
Queimadura	91,33	7,41	76,8; 105,87	52 (29,1)	
Atropelamento	96,02	5,12	85,99; 106,05	142 (12,9)	
Corpo estranho	57,67	1,31	55,11; 60,24	1 (1,8)	
Retorno	81,06	5,66	69,97; 92,15	76 (9,7)	

^a^Significativo se p-valor<0,050.

**Figura 1:** Curva de sobrevida estimada pelo método de Kaplan-Meier para o tempo de permanência entre os motivos de entrada, Belo Horizonte, 2015-2019 (n=10.742).

O modelo foi significativo p-valor<0,001. Houve diferenças dos riscos de morrer entre os motivos. Os riscos de morte por queda, intoxicação, motivo clínico, autoagressão ou atropelamento são 1,9, 1,9, 1,8, 3,1 e 1,5 vezes maiores em relação ao motivo de acidente (Tabela 3 e Figura 2). 

**Tabela 3:** Razões de risco (RR) e intervalos de confiança de 95% (IC95%) do motivo de entrada no hospital via regressão de Cox. Belo Horizonte, 2015-2019 (n=10.742)

Motivo de entrada	RR	IC95%	p-valor
Acidente	1	-	-
Queda	1,856	1,335; 2,581	<0,001
Agressão	1,358	0,790; 2,334	0,269
Intoxicação	1,925	1,059; 3,499	0,032
Motivo clínico	1,775	1,271; 2,480	0,001
Autoagressão	3,133	1,838; 5,341	<0,001
Queimadura	1,453	0,954; 2,212	0,082
Atropelamento	1,471	1,028; 2,105	0,035
Corpo estranho	0,250	0,034; 1,818	0,171
Retorno	1,205	0,816; 1,780	0,347

**Figura 2:** Riscos estimados pelo método de Cox para o tempo de internação entre os motivos de entrada, Belo Horizonte, 2015-2019 (n=10.742).

A menor proporção de falhas associada ao motivo de entrada por corpo estranho (1,8%) destacou a possível relação entre esse motivo e melhores resultados clínicos. Tal fenômeno sugeriu um quadro de menor gravidade ou menor risco de desfechos adversos.

A relevância do teste de log-rank, que indicou diferenças significativas nas curvas de sobrevida em relação aos diferentes motivos de entrada e à maior estimativa de tempo de internação para o motivo de atropelamento (96 dias), sublinha a necessidade a necessidade de abordagem personalizada para cada caso. A variação nos riscos de morte associados aos diferentes motivos (1,9, 1,9, 1,8, 3,1 e 1,5 vezes maiores) enfatizou a necessidade de avaliações e cuidados específicos para cada situação.

## Discussão

Ao analisar a prevalência das internações hospitalares de pessoas idosas, identificou-se que o sexo masculino foi predominante, o que evidenciou a maior vulnerabilidade do homem idoso, especialmente em situações envolvendo acidentes externos ou piora de condições clínicas crônicas. As faixas etárias corresponderam às pessoas idosas de 60-64 anos e 65-69 anos, possivelmente devido ao maior grau de independência e envolvimento em atividades físicas e sociais, o que pode aumentar a exposição a traumas. Observou-se que a maioria das internações ocorreu em Belo Horizonte, fato relacionado à localização do hospital de referência na capital mineira e ao contexto populacional de uma cidade com mais de 2,5 milhões de habitantes (11). Isso demonstrou que a oferta de serviços de saúde em Minas Gerais ocorre de acordo com o Plano Diretor de Regionalização da Saúde, atribuindo a cada região uma parcela de responsabilidade na assistência de acordo com a economia de escala e escopo para que se amplie a acessibilidade ao tratamento demandado. 

O estudo mostrou que 50,0% das pessoas idosas hospitalizadas permaneceram internadas em média 79,8 dias, dado que reforça a importância de estratégias voltadas à gestão eficiente do tempo de internação. A taxa de óbitos observada, de 11,7%, juntamente com os desafios clínicos destacados ao longo do acompanhamento, evidenciou a complexidade dos casos tratados e apontou para a necessidade de fortalecimento dos cuidados paliativos e do suporte emocional. Esses resultados revelaram o panorama que demanda esforços interdisciplinares para melhorar a qualidade de vida da população idosa, reduzir os custos associados à assistência hospitalar prolongada e oferecer alternativas de cuidado mais adequadas. 

Os resultados evidenciados trazem à tona uma série de implicações significativas para os serviços de saúde, especialmente ao considerar o aumento do tempo de permanência e a redução gradativa da taxa de sobrevida dos pacientes. O fato de 50,0% das pessoas idosas permanecerem internadas por média de 79,8 dias, com desvio-padrão de σ de 1,8 mês, apontou a duração média considerável de internação. Esse prolongamento pode acarretar custos elevados para os serviços de saúde, incluindo a utilização de recursos financeiros, a ocupação prolongada de leitos hospitalares e o aumento da demanda sobre a equipe médica (12,13).

A observação da taxa de óbitos de 11,7% e da censura de 88,3% para os demais pacientes evidencia a complexidade dos casos tratados. Esses indicadores sugerem um cenário clínico desafiador, no qual muitas pessoas não conseguem completar o acompanhamento necessário. Esse contexto pode estar associado à dificuldade em manter a população idosa sob cuidados intensivos por períodos prolongados, ressaltando a importância de estratégias voltadas aos cuidados paliativos e ao suporte emocional (14). 

Para os serviços de saúde, esses achados apontaram para a necessidade de implementar estratégias de gestão mais eficientes, que contemplem a redução do tempo de internação, a melhoria da qualidade de vida e a oferta de alternativas de cuidado à população idosa. A colaboração entre equipes multidisciplinares, a atenção aos fatores que influenciam a taxa de sobrevida e a otimização do uso dos recursos são essenciais para garantir o equilíbrio entre a eficácia clínica, a qualidade de vida do paciente e a sustentabilidade dos serviços de saúde (12,15).

A análise de riscos estimados pelo método de Cox, referente ao tempo de internação de acordo com os motivos de entrada, demonstrou a relevância da triagem eficaz no momento da admissão hospitalar. Essa prática permite a alocação mais adequada de recursos e a aplicação de intervenções direcionadas. Destaca-se que a qualidade de vida da pessoa idosa foi duas vezes maior às estratégias de cuidados de saúde específicas para os motivos de internação. Ao adaptar as abordagens de tratamento e prevenção, os serviços de saúde podem melhorar os resultados clínicos e contribuir para a melhor qualidade de vida das pessoas idosas, abordando às suas necessidades de maneira abrangente e eficiente (3,16,17). 

Este estudo ofereceu a perspectiva holística sobre os serviços de saúde e a qualidade de vida da população idosa, abordando aspectos como o tempo de internação, o risco de morte, o gênero, a faixa etária e o motivo de entrada. Esta abordagem multifacetada ressaltou a complexidade do cuidado às pessoas idosas e destacou a importância da intervenção integrada e personalizada para otimizar os resultados de saúde e a qualidade de vida nesse grupo demográfico. Tornou-se evidente a importância de implementar políticas públicas de prevenção de trauma e agravos clínicos na população idosa. 

Defende-se que, por meio da aplicação de medidas que promovam o envelhecimento saudável, a prevenção e o tratamento eficaz de doenças crônicas, além de campanhas de educação no trânsito, é possível alcançar resultados expressivos na melhoria da qualidade de vida da pessoa idosa. Destaca-se a importância de que os profissionais atuantes nesse contexto sejam continuamente capacitados para oferecer o cuidado e o tratamento mais qualificados à população idosa. 

## Data Availability

O banco de dados está disponível sob demanda dos parecerista, com justificativa plausível, uma vez que possuem dados sensíveis.
